# Careful Phenotypic Characterization of Tremor Phenomenology in a Patient with Spinocerebellar Ataxia Type 12-Tremor Features Do Not Match Those of Essential Tremor

**DOI:** 10.5334/tohm.889

**Published:** 2024-06-05

**Authors:** Weili Luo, Xiaosheng Zheng, Zhiru Lin, Wei Luo

**Affiliations:** 1 Department of Neurology, Municipal Hospital Affiliated to the Taizhou University, Taizhou, China; 2 Department of Neurology, The Second Affiliated Hospital, Zhejiang University School of Medicine, Hangzhou, Zhejiang, China; 3 Department of Neurology, Wenzhou Central Hospital, Wenzhou, Zhejiang, China

**Keywords:** Spinocerebellar Ataxia Type 12, PPP2R2B, essential tremor

## Abstract

**Background:**

The tremor characteristics of patients with spinocerebellar ataxia 12 (SCA12) are often likened to those in patients with essential tremor (ET); however, data are sparse, and videotaped tremor examinations are rare.

**Case Report:**

A 37-year-old woman with progressive hand and head tremors underwent genetic testing after conventional diagnostics failed to explain her symptoms. A *PPP2R2B* variation confirmed spinocerebellar ataxia type 12 (SCA12), a condition not previously considered because classical cerebellar signs were absent. The tremor characteristics of this patient differed in numerous respects from those seen in patients with ET.

**Discussion:**

Although often likened to ET, under careful scrutiny, the tremor characteristics observed in this patient with SCA12 were inconsistent with those typically seen in ET. Such discrepancies highlight the necessity of careful phenotyping for tremor disorders, particularly in familial cases. Recognizing the specific tremor phenomenology of SCA12 and distinguishing it from ET is crucial to avoid misdiagnosis and to guide appropriate management and familial counseling.

**Highlights:**

This report characterizes in detail an early-stage SCA12 patient initially misdiagnosed as essential tremor, underscoring the importance of nuanced clinical assessment and genetic testing in atypical tremor cases. Similar patients should be meticulously phenotyped to prevent misclassification and enhance our understanding of tremor pathophysiology.

## Background

The diagnosis of tremor disorders can be challenging. In this case study, we describe a female patient who exhibited tremors exclusively in the upper limbs and head, and who was ultimately diagnosed with spinocerebellar ataxia type 12 (SCA12). The tremor characteristics of patients with SCA12 are often likened to those in patients with essential tremor (ET); however, data are sparse, and videotaped tremor examinations are rare.

## Case report

A 37-year-old female patient of Han Chinese descent reported a three-year history of involuntary bilateral hand and head tremors, with simultaneous onset in both regions. The severity of these tremors had progressively increased, yet they remained localized to the hands and head without involving other body regions. Exacerbation of the tremors was noted in states of stress or anxiety, and the head tremor remained persistent in the supine position. The tremor could not be alleviated by alcohol intake. The patient sought medical attention for her symptoms because they increasingly impacted her ability to perform her daily activities.

**Figure 1:**
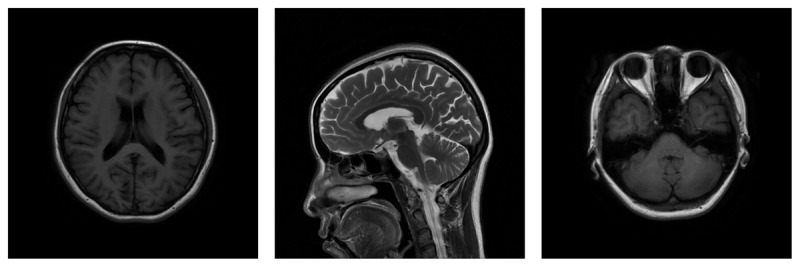
MRI shows no cerebral atrophy, no cerebellar atrophy, or other structural abnormalities.

Upon neurological examination (Video 1), there was a mild postural tremor involving the fingers rather than the wrist or elbow. On finger to nose maneuver, mild intention tremors were observed in both hands, and mild dysmetria on the left. There was a ‘no-no’ (horizontal) head tremor. The head tremor was irregular, and there was a slight torticollis with the head tilting to the right. However, there were no tremors in the mandible or the protruded tongue. The head tremor persisted when she was supine. Rapid alternating movement tests yielded normal results. Dysmetria was observed during the initial phase of the finger-nose-finger maneuver on both right and left sides. There were no signs of dysarthria. Ocular assessments revealed normal saccades and pursuit movements. There were no indications of bradykinesia, muscle rigidity, or hyperreflexia. Babinski signs were absent and no muscular weakness, sensory abnormalities, or signs of bladder or bowel disturbances were observed. A brain MRI revealed no cerebellar or cerebral atrophy, and no other structural abnormalities (Figure 1). Comprehensive laboratory evaluations, including thyroid function tests, and serum levels of ceruloplasmin, ferritin, and vitamin B12, were within normal limits.

**Video 1:** **Neurological Assessment Demonstrating Mild Postural and Intention Tremors with Preserved Proprioceptive Function.** This video demonstrates a mild postural tremor involving distal joints of both hands rather than involvement of the wrist or elbow. A head tilt is noted to the right, and head tremor is irregular. On finger to nose maneuver, intention tremor is evident, although worse on the left, where mild dysmetria briefly evident. Romberg’s sign is negative, indicating preserved proprioceptive function. Tandem gait abnormalities were not evident in this assessment.

**Figure 2:**
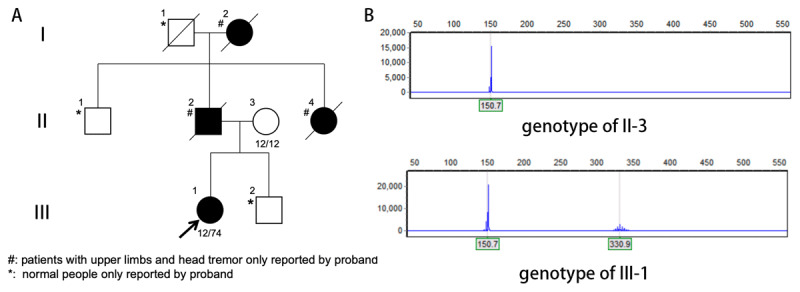
The patient’s family genetic profile. **(A)** Pedigree chart showing symptom occurrence and genetic status. **(B)** Capillary electrophoresis results with genotypes of II:3 (12/12) and III:1 (12/74).

The patient’s medical history revealed a familial pattern of tremor, with her father, grandmother, and aunt all having experienced similar symptoms of head and hand tremors (Figure 2A). The proband’s father (II–2) tragically died in a motor vehicle accident in his forties. The other family members (I–2 and II–4) lived normal lifespans and died in their seventies. Notably, none of these three deceased individuals received a formal medical diagnosis or treatment. Their clinical manifestations were largely restricted to hand and head tremor, akin to the symptoms observed in the proband. There was no report of other features typically associated with SCA12, such as gait impairment, dysarthria, bradykinesia, behavioral or psychiatric disturbances, or urinary abnormalities. Whole genome sequencing data revealed a CAG repeat expansion in the *PPP2R2B* gene. Subsequent sanger sequencing with capillary electrophoresis confirmed a repeat count of 74, falling within the range of full penetrance. In an effort to trace the genetic lineage of this variation, the proband’s mother underwent testing for CAG repeat expansion in *PPP2R2B*. The absence of this variation in her genome indicates a paternal inheritance of the condition, corroborating the patient’s familial history (Figure 2B) and the observed phenotypic manifestations within the paternal lineage of the family.

## Discussion

SCA12 is a late-onset autosomal dominant neurodegenerative disorder, primarily caused by CAG repeat expansions in *PPP2R2B* [1]. Initially described in individuals of German ancestry, the majority of reported SCA12 cases are from India, predominantly within the endogamous Agarwal ethnic group [2,3,4]. Han Chinese patients have also been reported with CAG repeat expansions ranging from 51–56, which is at the threshold of full penetrance [5,6]. The 74-repeat expansion observed in the proband of this study indicates that larger expansions are also present in the Han Chinese ethnic group.

Initial manifestation of SCA12 typically involves symmetric or asymmetric action tremor in the upper limbs that is often followed or accompanied by other movement abnormalities, such as head tremor, dysarthria, and gait ataxia. Medical consultation is often delayed for many patients, leading to diagnoses of tremor when other neurological signs are already evident. Achal et al. [7] described a case with a 47-repeat CAG expansion in *PPP2R2B* and a disease duration of four years, who exhibited no deficits other than head tremor. Similarly, we report here a 37-year-old female patient with a three-year disease duration, presenting with symptoms that superficially resembled ET. Given her positive family history, whole genome sequencing was applied and a repeat expansion in *PPP2R2B* was discovered. This expansion was previously undetected in a cohort of 323 ET patients [8].

The tremor characteristics of patients with SCA12 are often likened to those in patients with essential tremor (ET); however, data are sparse, and videotaped tremor examinations are rare. The tremor characteristics of this patient differed in numerous respects from those seen in patients with ET. Here we discuss several of these features that are not typical of ET. First, although head tremor is the most common cranial tremor in ET patients, with a prevalence ranging from 15% to 55%, it typically develops after the appearance of arm tremor. Conversely, a pattern starting with head tremor (or with simultaneous onset of head and limb tremor) is extremely rare [9]. Second, the observed postural tremor in our patient was characterized by mild tremor in the distal joints (fingers) rather than the more proximal (wrist and elbow) tremor that is typically seen in ET. In ET, the greatest amplitude of postural tremor usually occurs at the wrist or elbow, not at the metacarpophalangeal joints of the fingers [10,11]. Third, the head tremor exhibited was irregular, accompanied by mild torticollis and persisted in the supine position, which are features that are consistent with characteristics of dystonic tremor. The head tremor in ET typically resolves when the patient is supine [12,13].

Athough most SCA12 cases reported in larger-scale studies present with tremor as an initial symptom, and this is often likened to that seen in ET [14], our report thoroughly characterizes the tremor phenomenology in an early-stage SCA12 patient, noting differences in comparison to the tremor of ET. Future cases should be phenotyped meticulously, especially with respect to tremor phenomenology, to prevent misclassification of their clinical features as those typical of ET.
